# Impact of promoting longer-lasting insecticide treatment of bed nets upon malaria transmission in a rural Tanzanian setting with pre-existing high coverage of untreated nets

**DOI:** 10.1186/1475-2875-9-187

**Published:** 2010-06-28

**Authors:** Tanya L Russell, Dickson W Lwetoijera, Deodatus Maliti, Beatrice Chipwaza, Japhet Kihonda, J Derek Charlwood, Thomas A Smith, Christian Lengeler, Mathew A Mwanyangala, Rose Nathan, Bart GJ Knols, Willem Takken, Gerry F Killeen

**Affiliations:** 1 Biomedical and Environmental Thematic Group, Ifakara Health Institute, P.O. Box 53, Ifakara, Tanzania; 2 Department of Biological and Biomedical Sciences, Durham University, South Road, Durham, DH1 3LE, UK; 3 Vector Group, Liverpool School of Tropical Medicine, Pembroke Place, Liverpool, L3 5QA, UK; 4 Department of Zoology and Marine Biology, University of Dar es Salaam, P.O. Box 35064, Dar es Salaam, Tanzania; 5 DBL Centre for Health Research & Development, 57 Thorvaldensvej, Fredriksberg -C, DK 1870, Denmark; 6 Department of Public Health and Epidemiology, Swiss Tropical Institute, Socinstrasse 57, Basel, CH 4002, Switzerland; 7 Division of Infectious Diseases, Tropical Medicine & AIDS Academic Medical Center, F4-217, Meibergdreef 9, 1105 AZ, Amsterdam, The Netherlands; 8 Laboratory of Entomology, Wageningen University and Research Centre, P.O. Box 8031, 6700 EH, Wageningen, The Netherlands

## Abstract

**Background:**

The communities of Namawala and Idete villages in southern Tanzania experienced extremely high malaria transmission in the 1990s. By 2001-03, following high usage rates (75% of all age groups) of untreated bed nets, a 4.2-fold reduction in malaria transmission intensity was achieved. Since 2006, a national-scale programme has promoted the use of longer-lasting insecticide treatment kits (consisting of an insecticide plus binder) co-packaged with all bed nets manufactured in the country.

**Methods:**

The entomological inoculation rate (EIR) was estimated through monthly surveys in 72 houses randomly selected in each of the two villages. Mosquitoes were caught using CDC light traps placed beside occupied bed nets between January and December 2008 (*n *= 1,648 trap nights). Sub-samples of mosquitoes were taken from each trap to determine parity status, sporozoite infection and *Anopheles gambiae *complex sibling species identity.

**Results:**

Compared with a historical mean EIR of ~1400 infectious bites/person/year (ib/p/y) in 1990-94; the 2008 estimate of 81 ib/p/y represents an 18-fold reduction for an unprotected person without a net. The combined impact of longer-lasting insecticide treatments as well as high bed net coverage was associated with a 4.6-fold reduction in EIR, on top of the impact from the use of untreated nets alone. The scale-up of bed nets and subsequent insecticidal treatment has reduced the density of the anthropophagic, endophagic primary vector species, *Anopheles gambiae sensu stricto*, by 79%. In contrast, the reduction in density of the zoophagic, exophagic sibling species *Anopheles arabiensis *was only 38%.

**Conclusion:**

Insecticide treatment of nets reduced the intensity of malaria transmission in addition to that achieved by the untreated nets alone. Impacts were most pronounced against the highly anthropophagic, endophagic primary vector, leading to a shift in the sibling species composition of the *A. gambiae *complex.

## Background

In much of Africa, where malaria transmission levels are extremely high, substantial reductions in the intensity of transmission are required for even a modest reduction in human parasitaemia [1,2]. Over the past decade, a major malaria control strategy has been the use of insecticide-treated nets (ITNs), which are perhaps the best-evaluated and most cost-effective intervention for large-scale application [3-7]. The distribution programmes used in different countries have been as diverse as they have been numerous; but the goal to increase the coverage of both nets and insecticide levels has been common to all. In recent years, a number of success stories have emerged and the incidence of malaria has begun to decline in many regions of Africa [5-7].

The protective efficacy of ITNs results from both the physical barrier and the insecticidal action of the net. While it is intuitively clear that ITNs provide protection to individual users, what is less obvious is the impact of widespread ITN use at the community level. ITNs are able to reduce the density, feeding frequency and survival of mosquitoes [8-11] and wide-scale use can mediate protection of all community members, including the vulnerable portion without a bed net [12-15]. With moderate ITN coverage of the population, the 'mass effect' is at least as important as the personal protection provided to the user [12,14,16]. On the other hand, it has been suggested that ITN use could increase malaria risk for unprotected people by diverting mosquitoes away from users and concentrating their host-seeking efforts upon them [9,17,18]. Although theoretically possible, field studies have demonstrated that the beneficial impacts on malaria transmission outweigh any such inequitable biting burden for the unprotected [8,14,19,20].

The current study is a retrospective analysis examining the impact of introducing a longer-lasting insecticide treatment into a setting with pre-existing high coverage of largely untreated nets. Two study villages in rural Tanzania experienced year-round, hyperendemic malaria transmission in the 1990s [21]. By the early 2000s, high coverage of untreated nets was achieved using a cost-sharing scheme for subsidisation and promotion [22]. More recently, national-scale subsidisation programmes co-packaged longer-lasting insecticide treatment kits with all bed nets manufactured in Tanzania and promoted their use from 2004 onwards [23,24]. The current study investigated whether the treatment of bed nets with longer-lasting insecticide produced any further impact on the intensity of malaria transmission beyond the 4.2-fold reduction observed from the use of untreated nets alone [22]. In order to examine changes in the entomologic inoculation rate (EIR) and the biodemographic profile of vector mosquitoes, their biting-density, sporozoite prevalence and survival was estimated in these same two villages throughout 2008. Results were compared with data collected from the same villages during 1990-94 before bed net use was common and during 2001-03 after high coverage of untreated bed nets had been achieved.

## Methods

### Study area

**Figure 1:**
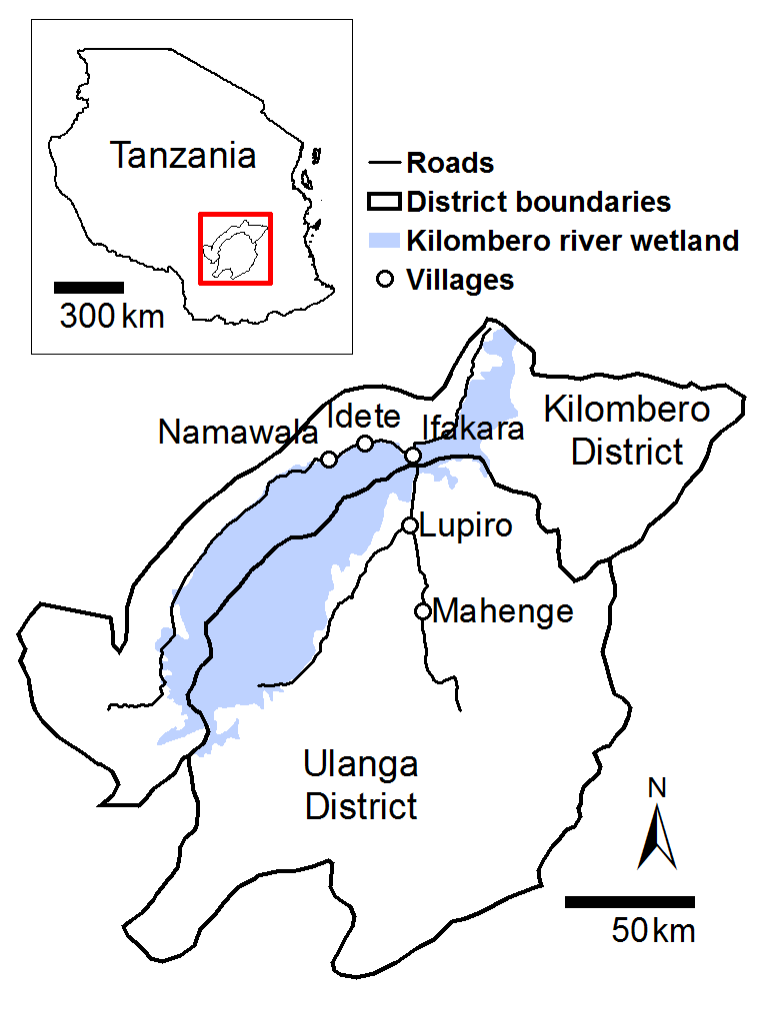
**Kilombero and Ulanga districts (8.1°S and 36.6°E) in Tanzania showing Namawala and Idete villages**.

The study was conducted in Namawala and Idete villages, located in the Kilombero Valley (8.1°S and 36.6°E) in south-eastern Tanzania (Figure 1). These communities experience hyper endemic malaria transmission [25], mostly transmitted by large populations of mosquitoes from the *Anopheles gambiae sensu lato *complex (Diptera: Culicidae) [26,27]. In this area, this species complex is represented by two morphologically identical, but behaviourally distinctive, sibling species: *A. gambiae sensu stricto *(hereafter referred to as *A. gambiae*) and *Anopheles arabiensis*. A third, locally important vector species is *Anopheles funestus*. The ecosystem is dominated by a low lying river valley, 150 km long and up to 40 km wide, which is inter-dispersed with villages and rice farms. Annual flooding occurs during the rainy season (December - May) when large tracts of aquatic habitat suitable for immature mosquitoes are formed.

The epidemiology of malaria in the study villages has been well characterised over the past 15 years [e.g. [21,22,28-34]. Extremely high transmission intensities were recorded during the 1990s [21]. Since 1997, various cost-sharing schemes for subsidizing and promoting bed nets, as well as home insecticide treatment kits have been implemented in an effort to alleviate the malaria burden. The crux of the various programmes has been the generic branding of recommended nets and insecticides products which were sold in line with a price-fixing scheme that reflected a public subsidy (34% of retail value at US $5). To improve access to vulnerable pregnant women and infants, a further subsidy (17% of retail value) was provided through the use of a voucher scheme. The pregnant women and mothers of young children who attended antenatal or immunisation clinics were entitled to a discount voucher.

The initial pilot programme, KINET, distributed bed nets within the Kilombero Valley and achieved remarkably high bed net coverage of all community members [16,22,25,35-37]. Although all KINET distributed bed nets were pre-treated with 20 mg/m^2 ^deltamethrin [37], by 2001, insecticide levels had fallen below 5% and most nets were in poor condition containing many holes [22,38]. Various national-scale distribution programmes have been implemented, commencing with PSI's Social Marketing of Insecticide Treated Nets (SMITN) programme which was run at a regional-scale during 1998-2000 and a national-scale during 2000-02 and promoted the use of nets and standard insecticide treatment kits (KO Tab, Icon^® ^and Fendona). The sequential programme was SMARTNET from 2002, which the Tanzanian National Voucher Scheme (TNVS) was built upon in 2004. SMARTNET ensured that all bed nets manufactured in Tanzania were co-packaged with longer-lasting insecticide treatment kits, which were registered for use from 2004 onwards (Initially: KO Tab 123, target dose: 25 mg deltamethrin/m^2 ^[39,40]; and from 2008: Icon^® ^MAXX, target dose: 50 mg lambda-cyhalothrin/m^2 ^[41]).

### Experimental design

In each village, 72 households were randomly selected for mosquito collection from the household list of the Ifakara Health Institute (IHI) Demographic Surveillance System (DSS) [25]. Each house was visited once a month (6 houses/day, 4 days/week and 3 weeks/month) over a period of 12 months (January to December 2008). Mosquitoes were collected inside houses using one CDC light trap that was placed beside a person sleeping underneath a bed net and left to run for 12 hours (7 pm - 7 am) [42]. The light trap, fitted with an incandescent bulb, was placed 1 - 1.5 m above the floor and close to the feet of a bed net occupant. The bed net provided to the participating households was a new long-lasting insecticidal bed net (Olyset, A to Z Textile Mills Ltd., Tanzania). Although permethrin-treated bed nets exhibit modest excito-repellency, they have surprising little effect on the relative efficiency of light traps when compared with untreated bed nets [22,43].

### Mosquito sampling and processing

After each night of trapping, all mosquitoes were morphologically identified to sex and species then visually classified as being unfed, partially fed, fully fed or gravid [27,44]. Throughout the study, sub-samples of up to 9 individual mosquitoes were taken from each trap to determine parity status, sporozoite infection and sibling species identity. Mosquito survival was assessed using ovarian dissection for parity (parous versus nulliparous) [45], sporozoite infection was determined using ELISA [46] and the sibling species identity of the *A. gambiae *complex specimens were determined using PCR [47]. Prior to molecular analysis, individual mosquitoes were stored at -20°C in micro centrifuge tubes containing a small amount of silica drying agent separated from the mosquito by a thin layer of cotton.

### Housing and climatic conditions

The physical structure and size of eave openings for each randomly selected house was recorded directly. The use of bed nets and cattle ownership was estimated by the IHI DSS [25] during an annual survey of all households in the study villages. Each household head was asked: 1) how many people slept in the house, 2) how many people slept under a bed net the previous night, 3) how many bed nets the household had, 4) how many bed nets were treated, 5) in which month and year was the insecticide applied to each treated bed net and 6) how many head of cattle does the household own.

Rainfall data was collected on the nearby Kilombero Agricultural Training and Research Institute (<12 km from Idete village). The hourly variation in temperature was recorded using a data logger (Tinytag TV-1500, Gemini Data Logger, UK) placed inside a local house in nearby Lupiro village [22].

### Statistical analysis

Indoor mosquito sampling with CDC light traps is considered to be proportionally representative of true adult exposure [22,42,48,49] so the biting rate (B) was not adjusted for the number of household inhabitants. To account for the lower efficiency of CDC light traps relative to human landing catch, which we consider to be equivalent to the exposure of an unprotected person lacking a net, the biting rate was calculated by dividing the number of mosquitoes caught by species-specific relative efficiency, being 0.30 for the *A. gambiae *complex, 0.68 for *A. funestus *and 0.59 for *Culex *spp. [22].

The annual EIR was calculated using the equation: EIR = S × B × 365 [1,2,21,22]. Where, S (sporozoite prevalence) = no. of sporozoite positive mosquitoes/no. of mosquitoes tested, and B (biting rate) = no. of mosquitoes collected/(no. of trap nights × species-specific relative efficiency). The biting rate was calculated as an absolute mean opposed to the William's mean [33]. This approach provides a more realistic representation of the true total exposure of humans to malaria infection, as the majority of transmission events are due to a fraction of vectors that are commonly associated with high densities in over-dispersed data [50,51].

The above approach is consistent with previous surveys of these villages allowing these data to be directly compared with previous estimates [21,22,30,33]. Comparable S, B and EIR values were available from previous surveys in 1990-94 [22,29-34] and 2001-03 [22]. Each sampling period analysed represented a different phase of bed net coverage: 1990-94 preceded any substantive uptake of bed nets in this area [52] while 2001-03 followed a long-term programme of bed net promotion resulting in wide-scale use of untreated nets [22] and 2008 saw significant uptake of longer-lasting insecticide treatment kits as a result of national-scale efforts to promote them. Bed net coverage was calculated as the proportion of people sleeping under a bed net on the previous night. The yes/no question asking if a bed net had even been treated was answered by all of the respondents, but only some of the positive respondents were able to delineate the time-frame for bed net treatment. As such, the proportion of treated bed nets was estimated by weighting the proportion of bed nets that had ever been treated by the proportion that were treated within the past 12 months.

The temporal change in annual village-level S, B, EIR, proportion parous and *A. gambiae *complex sibling species composition was analysed using generalised linear models (GLMs) with a categorical explanatory variable for study period. For the response variables B and EIR, the GLM used a negative binomial distribution and a log link function. For the variables S, proportion parous and sibling species composition, the GLM used a binomial distribution and a logit link function. For these parameters, comparing annual village-level means over entire calendar-years averaged out short term temporal and spatial heterogeneity and thereby the analysis focused on long-term changes in these entomological parameters. As sporozoite prevalence is a property of entire communities rather than individual sampled houses [53], the village-level was considered to be the experimental unit (*n *= 2) for which B and S were both estimated as means of all samples from all houses over the entire year.

The construction of houses is known to influence the indoor densities of mosquitoes [54,55] so an additional analysis was conducted to examine this effect at the household level. The effect of closing eaves on the annual biting rate of Anopheline mosquitoes (B) for 2008 was analysed using a generalised linear mixed model (GLMM) [56] with eaves as a fixed factor and household and month as a random factor to account for repeated sampling. This GLMM model used a negative binomial distribution and a log link function. All analyses were conducted using the *R *package V2.9.1.

### Ethics

Ethical approval for the study was obtained from the IHI Institutional Review Board (IHRDC/IRB/No. A-32) and the Medical Research Coordination Committee of the National Institute for Medical Research (NIMR/HQ/R.8a/Vol. IX/764) in Tanzania. When the study commenced, permission was obtained from each household owner who was informed about the potential risks and benefits of participation both orally and via provision of a written pamphlet. After consenting, the household head signed an informed consent form stating their willingness to participate in the study.

## Results

During the 12 month survey 1,648 CDC light trap nights of sampling were conducted. A total of 97,437 female mosquitoes were caught, of which 98.5% were unfed and thus considered to have been mostly caught in the act of host-seeking. Of these mosquitoes, 30.9% were *A. gambiae *complex (*n *= 30,111) comprising 85.8% *A. arabiensis *and 14.2% *A. gambiae senso stricto *(*n *= 2,924 PCR amplifications). The remaining mosquitoes were 2.0% *A. funestus *(*n *= 1,950), 62.0% *Culex *spp (*n *= 60,442), 2.4% *Mansonia *spp (*n *= 2,302) and 2.7% other species including *Aedes *and *Coquillettidia *spp (*n *= 2,605).

**Figure 2:**
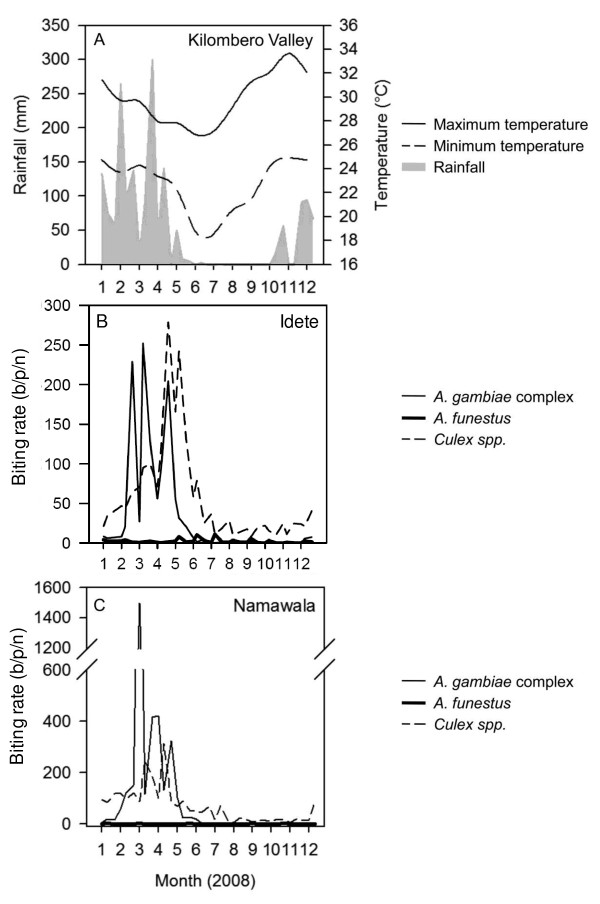
**Weekly rainfall and temperature throughout the Kilombero Valley (A) and nightly biting rate of mosquitoes for Idete (B) and Namawala (C) estimated using the mean weekly CDC light trap catch adjusted by dividing by species-specific relative efficiency of 0.3, 0.68 and 0.59 for *Anopheles gambiae *complex, *A. funestus *and *Culex *spp. respectively [see **[22]**]**.

The density of mosquitoes was temporally and spatially heterogeneous with the bulk of the mosquitoes (97.4%) being caught between January and May. During this time there were multiple short-term peaks of mosquito emergence that occurred after rainfall (Figure 2). Members of the *A. gambiae *complex dominated the biting burden while *A. funestus *contributed to only 3% of the total *Anopheles *bites occurring in these two villages (Table 1).

**Table 1:** The estimated malaria transmission intensity attributable to the *Anopheles gambiae *complex and *Anopheles funestus*, computed for each village separately and the 2008 overall average

Species	Idete	Namawala	Overall
**Sporozoite prevalence (S; %)**			
*A. gambiae *complex	0.22	0.33	0.32
*No. tested by ELISA*	*1,858*	*3,148*	*5,006*
*A. funestus*	2.13	<0.09	1.71
*No. tested by ELISA*	*422*	*105*	*527*
**Biting rate (B; b/p/n)**			
*A. gambiae *complex	33.51	89.66	60.90
*A. funestus*	2.40	1.05	1.74
*No. of trap nights*	*916*	*732*	*1,648*
**Entomological inoculation rate (EIR; ib/p/y)**			
*A. gambiae *complex	26.3	124.8	71.1
*A. funestus*	18.7	0.3	10.8
*Total*	*45.0*	*125.1*	*81.9*
**Proportion parous**			
*A. gambiae *complex	0.45	0.54	0.51
*No. dissected*	*353*	*736*	*1,089*
***A. gambiae *complex sibling species proportion**			
*A. arabiensis*	0.87	0.84	0.86
*A. gambiae*	0.12	0.15	0.14
*No. PCR amplifications*	*1,481*	*2,599*	*4,080*
**Bed net usage (%)**^**a**^			
Untreated	41.0	47.3	44.8
Treated	47.0	46.6	46.8
Overall	87.9	94.0	91.5
*No. of bed net users*	*4,112*	*6,551*	*10,663*

S = no. of sporozoite positive mosquitoes/no. of mosquitoes tested

B = no. of mosquitoes collected/no. of trap nights/calibration factor of 0.30 for *A. gambiae *complex and 0.68 for *A. funestus*; and EIR = S × B × 365

^a ^Calculated as the percentage of people who slept under a bed net the previous night; bed nets were considered to be treated if insecticide had been applied in the previous 12 months

The sporozoite prevalence of *A. funestus *(1.71% infected, *n *= 527) and *A. gambiae *(1.18% infected, *n *= 507) were not different (χ^2 ^= 0.197, *p *= 0.656). Whereas, the sporozoite rate for *A. arabiensis *(0.16% infected, *n *= 3,116) was nearly 5-fold lower than both *A. funestus *(χ^2 ^= 24.29, *p *< 0.0001) and *A. gambiae *(χ^2 ^= 11.88, *p *= 0.0005). There was no difference in prevalence between villages for any of the species (*A. gambiae *complex: χ^2 ^= 0.57, *p *= 0.45; *A. funestus*: χ^2 ^= 2.28, *p *= 0.13).

The intensity of transmission experienced by unprotected people without a bed net provides the most direct indicator of community level transmission and protection. Overall, the EIR for non-users of bed nets was 81.9 infectious bites per person per year (ib/p/y, Table 1). Eighty-six percent of malaria transmission was attributable to the *A. gambiae *complex and 14% to *A. funestus*, with the majority of transmission (90%) occurring between January and May. Malaria transmission intensity was approximately 2-fold higher in Namawala than Idete, due to the high Anopheline biting rate in Namawala village.

**Figure 3:**
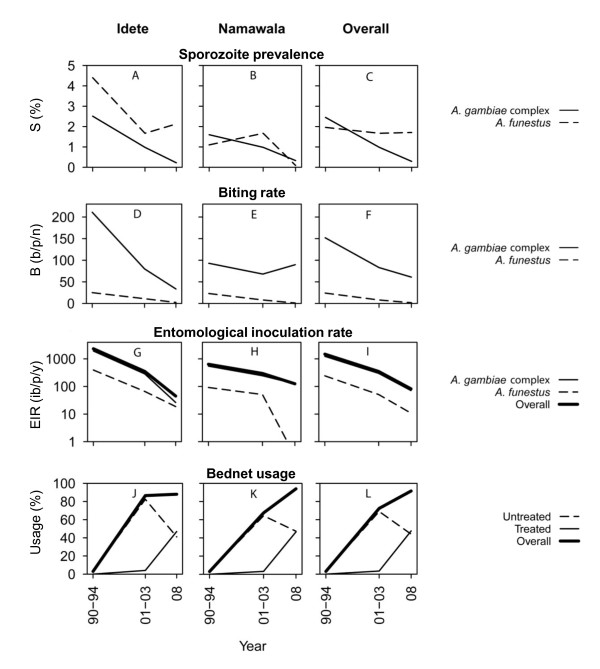
**Graphical comparison of the historical and recent estimates of sporozoite prevalence (A-C), biting rate (D-F) and entomological inoculation rate (G-H) for *Anopheles gambiae *complex and *Anopheles funestus *and overall**. The corresponding bed net usage recorded for the same areas and time periods (J-L) demonstrate the negative association between bed net usage and EIR. Note: Data for 1990-94 was from [29-34,52] and 2001-03 was from [22]. The B and EIR values for 1990-94 were recalculated by [22].

Similar entomological surveys using light traps were conducted in the same villages between 1990 and 1994 (before wide-spread bed net use) and again between 2001 and 2003 (after high coverage of untreated nets had been achieved), allowing a comparison of current malaria transmission intensities with historical rates. In 2008, bed net coverage levels were extremely high with 91.5% of the population sleeping under a net the previous night and 46.8% sleeping under an ITN. Since 1990-94 there has been an 8.4-fold reduction of the sporozoite prevalence of the *A. gambiae *complex, but surprisingly little change in the sporozoite prevalence of *A. funestus *(Table 2, Figure 3). The biting rate of the *A. gambiae *complex has reduced by 2.5-fold and for *A. funestus *by 13-fold. Between 1990-94 and 2001-03 the EIR was reduced by 4.2-fold [22] and between 2001-03 and 2008 by a further 4.6-fold. Thus, compared with the exposure of non-users in 1990-94 by 2008 there had been an 18-fold (95%) overall community level reduction in transmission intensity for non-users of bed nets. Considering that users of bed nets receive both personal and community level protection, the exposure of bed net users was calculated by adjusting for personal protection from 40% of bites for an untreated net user and 70% of bites for an ITN user [57]. As such, users of untreated nets probably experienced a 30-fold (97%) reduction and users of ITNs experienced a 60-fold (98%) reduction. In 2008, the mean EIR of an average community member calculated as an average weighted according to the recorded bed net use (Table 1) was 33.9 ib/p/y.

**Table 2:** Differences in the historical and recent estimates of sporozoite prevalence (S), biting rate (B) and entomological inoculation rate (EIR) for the *Anopheles gambiae *complex, *Anopheles funestus *and overall

Year	**OR**^**a **^**or RR**^**b **^**[95% CI]**	*p *value
***Sporozoite prevalence (S)***^**a**^		
***A. gambiae *complex**		
1990-1994^c^	1.00	NA
2001-2003^d^	0.450 [0.392, 0.518]	<0.0001
2008	0.128 [0.075, 0.218]	<0.0001
***A. funestus***		
1990-1994^c^	1.00	NA
2001-2003^d^	0.716 [0.609, 0.842]	<0.0001
2008	0.735 [0.377, 1.432]	0.366
**Overall**		
1990-1994^c^	1.00	NA
2001-2003^d^	0.530 [0.477, 0.589]	<0.0001
2008	0.185 [0.122, 0.281]	<0.0001
		
***Bites per person per night (B)***^**b**^		
***A. gambiae *complex**		
1990-1994^c^	1.00	NA
2001-2003^d^	0.486 [0.241, 0.983]	0.044
2008	0.405 [0.200, 0.821]	0.012
***A. funestus***		
1990-1994^c^	1.00	NA
2001-2003^d^	0.396 [0.233, 0.673]	0.0006
2008	0.072 [0.024, 0.214]	<0.0001
**Overall**		
1990-1994^c^	1.00	NA
2001-2003^d^	0.474 [0.250, 0.899]	0.022
2008	0.359 [0.188, 0.686]	0.0019
		
***Entomological inoculation rate (EIR)***^**b**^		
***A. gambiae *complex**		
1990-1994^c^	1.00	NA
2001-2003^d^	0.214 [0.075, 0.612]	0.004
2008	0.061 [0.021, 0.176]	<0.0001
***A. funestus***		
1990-1994^c^	1.00	NA
2001-2003^d^	0.241 [0.052, 1.100]	0.066
2008	0.039 [0.008, 0.188]	<0.0001
**Overall**		
1990-1994^c^	1.00	NA
2001-2003^d^	0.218 [0.089, 0.531]	0.0008
2008	0.057 [0.023, 0.141]	<0.0001

Village-level data were compared with a generalise linear model (GLM) using a binomial distribution with identity logit link for S and a negative binomial distribution with log link for B and EIR.

^a^Odds Ratio,

^b^Relative Rate,

^c^[29-34], ^d ^[22]

Temporal changes in malaria transmission intensity may be mediated by changes in a range of fundamental biological determinants of the mosquito population including the age distribution or species composition of the mosquito populations. Wide-spread bed net use appears to have mediated a drop in vector survival rates which was subsequently followed by population decline of highly anthropophagic species particularly once insecticide treatment became common. Overall, the proportion of parous mosquitoes has decreased from 0.62 in 1990-94 [30,31] to 0.43 in 2008 (Table 3). The proportion of *A. gambiae *among the complex decreased from 0.46 in 1990-94 [29,30,34] to 0.14 in 2008 (Table 3). During the initial six years of bed net use (commencing 1997) the parity rate of the mosquitoes declined, but at this time the proportions of *A. gambiae *and *A. arabiensis *remained fairly constant. By the time insecticide coverage had increased in 2008, additional reductions in the parity rate were very modest, but the population density of *A. gambiae *declined relative to that of *A. arabiensis*.

**Table 3:** The parity and sibling species composition of the *Anopheles gambiae *complex populations in averaged across Idete and Namawala villages

Year	Proportion (n/total)	Odds ratio [95% CI]	*p *value
***Parity***			
1990-1994^a^	0.62 (9,690/15,541)	1.00	NA
2001-2003^b^	0.47 (2,995/6,372)	0.535 [0.504, 0.568]	<0.0001
2008	0.51 (448/641)	0.422 [0.372, 0.478]	<0.0001
			
***Sibling A. gambiae sensu stricto***			
1990-1994^a^	0.46 (101/220)	1.00	NA
2001-2003^b^	0.38 (60/157)	0.812 [0.532, 1.240]	0.336
2008	0.14 (584/4080)	0.196 [0.148, 0.260]	<0.0001

^a ^[29-34], ^b ^[22]

The widespread use of ITNs placed a high level of stress on vectors that are highly endophagic and dependent on humans for blood [58], mediating a 79% reduction in *A. gambiae *density (69.9 b/p/n in 1990-94 versus 8.4 b/p/n in 2008). The reduction in *A. gambiae *density was presumably mediated by reduced survival. The proportion of parous *A. gambiae*, was very low at 0.39 (*n *= 76). On the other hand, bed net use has not had the same level of impact on the exophagic, zoophagic *A. arabiensis*, the density of which was only reduced by 38% since 1990-94 (82.0 b/p/n in 1990-94 versus 51.6 b/p/n in 2008). The proportion of parous *A. arabiensis *was 0.45 (*n *= 466) and higher than *A. gambiae*, but the difference in parity between the sibling species was not significant (χ^2 ^= 0.730, *p *= 0.393). The change in the composition of the *A. gambiae *complex resulted in 39% of transmission being attributable to *A. arabiensis *(calculated by adjusting both the sporozoite prevalence and biting rate), with 47% due to *A. gambiae *and 14% due to *A. funestus*. For the previous sample periods, we were unable to calculate the proportion of transmission attributable to the sibling species of the *A. gambiae *complex because species-specific sporozoite prevalence data was unavailable.

When interpreting the decline in malaria transmission over time, it is important to acknowledge other factors, such as increases in population density [59,60] or environmental changes [61-63], which can contribute to declines in malaria transmission. The population has increased dramatically from ~1,000 people per village in 1990-94 [52] to 4,673 people in Idete and 6,970 in Namawala in 2008. In 1990-94 there was an absence of cattle in the villages [52], but by 2008 there 306 head of cattle in Idete and 6,667 in Namawala. House construction may affect mosquito densities and consistent with published literature [54,55] the biting rate of the *A. gambiae *complex (Relative Rate (RR) [95% Confidence Intervals (CI)] = 0.689 [0.539, 0.882], *p *= 0.005) and *A. funestus *(RR [95% CI] = 0.858 [0.776, 0.948], *p *= 0.005) was lower in houses with closed eaves. The randomly selected houses during 2008 were built from clay-fired bricks (58%), mud (38%), grass thatch (2%) or clay unfired bricks (2%), with roofing of grass thatch (53%) or corrugated iron (47%). The majority (71%) had open eaves with a mean eave size of 14.6 ± 0.7 cm. In 1990-94, all of the houses were constructed from mud walls with thatched roofs [52], but importantly the portion of houses with open eaves was similar [54,55]. Although it rained heavily during 2008 (Figure 2a; total annual rainfall 2,401 mm) this would only have served to inflate the observed EIR values and provides further support to the results.

## Discussion

The study showed that in a setting where coverage of untreated bed nets was already high, the addition of longer-lasting insecticide treatment of bed nets was associated with a further 4.6-fold reduction in malaria transmission. This was in addition to the 4.2-fold reduction already associated with the use of untreated nets alone. Overall, an 18-fold reduction in transmission relative to the historical norms was recorded, demonstrating that combining the impacts of bed nets and insecticide, at high coverage rates, has a multiplicative effect along a linear scale or additive along a log scale [64,65].

The bed net distribution programmes which this area has been included in (the local pilot KINET and the various national-scale programmes) were based on a cost-sharing scheme that supported a commercial ITN distribution system combined with targeted subsidies for the most vulnerable community members. The programme is cost-effective [23] and was successful at achieving 91.5% use of bed nets by all community members, rather than just target groups, by 2008 (Table 1). Further, 46.8% of people slept under bed nets that were treated with longer-lasting insecticide within the last 12 months. It is important to note that the use of bed nets in the study villages is higher than the national average (40.8%) [66] since coverage was high before the national-scale programmes commenced.

The ITN coverage has reached the theoretical threshold (35 - 65%) required to mediate community level suppression of malaria mortality and morbidity [12]. The community-wide entomological impacts observed are consistent with epidemiological outputs in Idete village, where ITN use protected non-users from anaemia and splenomegaly [16]. Similarly, previous trials in sub-Saharan Africa have demonstrated a reduction in malaria mortality and/or morbidity of unprotected children who reside in or near clusters of households with high ITN use [14,15,19]. This study reflects the impact of ITN use on transmission under programmatic conditions where distribution is uneven, with protected and unprotected people inter-dispersed throughout the villages.

A substantial reduction in the intensity of malaria transmission, or EIR, was most likely associated with the ability of both untreated and treated nets to confer protection to the wider community, not just individual users. The EIR is the product of mosquito biting-densities (B) and the sporozoite rates (S) and both were reduced in the survey villages. The estimate of a reduced EIR by 95% for non-users is similar to previous estimates (90 - 94%) for ITN trials in Africa [67,68]. The most obvious mechanisms through which ITNs could have reduced vector density and survival are mortality when attacking an occupant of a ITN [8,67-69] and longer and more hazardous searches for blood-meals [70]. Regarding the sporozoite prevalence of the *A. gambiae *complex, this may have declined as a result of the reduced proportion of *A. gambiae *relative to *A. arabiensis*, since the sporozoite prevalence of *A. arabiensis *is consistently lower than that of *A. gambiae*. Other mechanisms that may also have contributed are the diversion of mosquitoes to alternative hosts [20,71,72], reduced feeding frequency [9,73] or reduced survival [67,68]. Surprisingly, the sporozoite prevalence of *A. funestus *did not change over time and in 2008 the sporozoite prevalence of *A. gambiae *was not different to *A. funestus*, suggesting that the sporozoite prevalence of *A. gambiae *had not changed with time either. The discrepancy between reduced mosquito survival corresponding with minimal impact on sporozoite prevalence may possibly be due to manipulation of infected mosquitoes by malaria parasites to extend their lifespan and increased feeding frequency [74].

With only two village-scale experimental unit replicates tracked through three time periods, the greatest limitation of this study is that it is essentially descriptive and observational. This study is a retrospective analysis of non-experimental data and, therefore, represents plausible rather than probable evidence [75] of community level suppression of transmission. It was not possible to contrast the results of this study with a control site where bed nets remained untreated, since preventing people from accessing ITNs would have been ethically inappropriate. Nonetheless, clear changes to the EIR and the bio-demographic profile of the vector species have been associated with the introduction of bed nets and insecticide treatments.

The biodemographic profile of mosquito populations may have also been altered by changes in land-use or landscape ecology [61-63]. With time, the population and geographic size of the villages has increased. During this process, some of the remote farming regions from 1990-94 have been urbanized. Regardless the general layout of the villages remains similar with a densely populated town centre and many people still residing in rural farming regions on the outskirts. During each study period, mosquito sampling was conducted in both farming and town houses, accounting for some of the biases caused by changes in landscape ecology over time. The highest densities of mosquitoes were captured in the farming regions where houses are constructed in close proximity to the larval habitats found in rice paddies. However, the observed reduction in EIR was much stronger than what would be expected due to changes in population size alone, as increases in population density can only mediate an equivalent reduction in transmission intensity [59,60]. Improvements in socio-economic status could also have contributed to the decreased risk of malaria transmission [76-78]. In general the socio-economic status of the villages has improved since 1990-94, as indicated by changes in house construction, a commonly used proxy measurement [79].

The introduction of bed nets, enforced with insecticides, had a stronger impact on the density and survival of the anthropophagic, endophagic *A. gambiae *than its zoophagic, exophagic sibling species *A. arabiensis*. The change in the proportional biting of the sibling species was not attributable to competitive displacement of *A. gambiae *by *A. arabiensis *[80], but merely a more severe reduction in *A. gambiae *density and survival. It is clear that the *A. arabiensis *population has been stressed by the use of ITNs, but this species may have adapted by either taking a higher portion of blood-meals from animal sources [81-83], or by biting earlier in the night when humans are outdoors and unprotected [84,85]. Similar shifts in sibling species composition due to selective pressure of domestic insecticide interventions were previously recorded for *A. gambiae *relative to *A. arabiensis *in Kenya [86,87] following sustained ITN use and for *A. funestus *relative to *Anopheles rivulorum *and/or *Anopheles parensis *in South Africa, Kenya and Tanzania following indoor-residual spraying [88,89]. Consequently, there is need for additional vector control tools that target exophagic, zoophagic vectors, such as *A. arabiensis*, to be integrated into existing malaria control programmes; for example zooprophylaxis [70], insecticide treated cattle [90], outdoor resting traps [91], or push-pull strategies [92].

## Conclusion

Insecticide treatment of nets reduced the intensity of malaria transmission in addition to that achieved by the untreated nets alone. Overall, an 18-fold (95%) community level reduction in transmission intensity was recorded for non-users of bed nets. These results clearly demonstrate that vector control in rural areas with high densities of mosquitoes is possible. The predominantly polyester-based net technologies currently used in these villages may be improved upon and further reductions are possible since the "catch up" programmes for free polyethylene-based long-lasting insecticidal bed net distribution commenced in late 2009. Impacts of insecticide-treated net use were most pronounced against the highly anthropophagic, endophagic primary vector, leading to a shift in the sibling species composition of the *A. gambiae *complex. Since almost 40% of transmission is now attributable to the exophagic, zoophagic *A. arabiensis*, additional vector control tools that specifically target this cryptic sibling species need to be integrated into existing malaria control programmes.

## Competing interests

The authors declare that they have no competing interests.

## Authors' contributions

TLR designed the study, supervised the mosquito sampling protocol, performed the data analysis and wrote the first draft of the manuscript. DWL implemented the longitudinal mosquito sampling protocol and assisted with data entry and analysis. DM and BC performed the molecular analysis of mosquito samples. JK supervised all aspects of mosquito sampling during all three study periods. JDC, TAS and CL designed and managed the collection of data during the 2001-03 sampling period. MAM and RN coordinated the collection of demographic and bed net usage data. BK and WT contributed to the study design and interpretation of the results. GFK contributed to the study design, data analysis and interpretation and drafting of the manuscript. All authors have read and approved the final manuscript.
